# Save the ICU and save lives during the COVID-19 pandemic

**DOI:** 10.1186/s40560-020-00456-1

**Published:** 2020-06-15

**Authors:** Japan ECMOnet for COVID-19, Ryuzo Abe, Naofumi Bunya, Tomoyuki Endo, Yuji Fujino, Kensuke Fujita, Kenji Fujizuka, Yoshihiro Hagiwara, Jun Hamaguchi, Yoshitaka Hara, Eiji Hashiba, Satoru Hashimoto, Noriyuki Hattori, Kota Hoshino, Shinichi Ijuin, Takanari Ikeyama, Shingo Ichiba, Wataru Iwanaga, Yoshiaki Iwashita, Masafumi Kanamoto, Hitoshi Kaneko, Kaneyuki Kawamae, Toru Kotani, Yasuaki Koyama, Keibun Liu, Tomohiko Masuno, Naoto Morimura, Tomoyuki Nakamura, Masaki Nakane, Michitaka Nasu, Osamu Nishida, Masaji Nishimura, Kanae Ochiai, Takayuki Ogura, Shinichiro Ohshimo, Keisuke Oyama, Junichi Sasaki, Ryutaro Seo, Takeshi Shimazu, Nobuaki Shime, Keiki Shimizu, Hiroyuki Suzuki, Shuhei Takauji, Shinhiro Takeda, Ichiro Takeuchi, Mumon Takita, Hayato Taniguchi, Nobuaki Shime

**Affiliations:** 1 Kawaguchi Heart and Lung Hospital, 1-1-51 Maekawa-cho, Kawaguchi, Saitama Prefecture 333-0842 Japan; 2 Department of Emergency and Critical Care Medicine, Graduate School of Biomedical & Health Sciences, Hiroshima University, 1-2-3 Kasumi, Minami-ku, Hiroshima, 734-8551 Japan

## Abstract

Appropriate critical care delivery for Coronavirus disease 2019 (COVID-19) is a cornerstone in saving lives. Earlier publications worldwide demonstrate higher mortality among patients receiving mechanical ventilation in intensive care units during “surges” in the number of cases. In contrast, lower mortality outcomes are evident in Japan using CRISIS [CRoss Icu Searchable Information System] data by the national registry, Japan ECMOnet for COVID-19. This highlights the need for scientific analysis of the medical factors contributing to high survival rates and social factors associated with low case “surges,” to gain insight into protective strategies for possible coming waves in the COVID-19 pandemic.

The Coronavirus disease 2019 (COVID-19) pandemic is a novel challenge for critical care systems worldwide. Appropriate critical care delivery for acute respiratory failure is a cornerstone in saving the lives of patients. A recent publication of JAMA [1] reports a large-scale observational study describing the clinical characteristics and outcomes of patients with COVID-19 in the New York City area. Among patients who were discharged or who died (*n* = 2634), 373 (14%) were treated in the intensive care unit (ICU), and 320 (12%) received invasive mechanical ventilation. Surprisingly, 88% of patients died while receiving mechanical ventilation. Similarly, results from a national registry in the UK [2] indicate that 62% of patients with confirmed COVID-19 and requiring advanced respiratory support in the ICU (2175/3508) died. Moreover, a report from the Lombardy region of Italy reported that among 1591 patients infected with SARS-CoV-2 admitted to ICUs, 26% died, 16% were discharged, and 58% were still in the ICU [3]. These high mortality rates could be partly explained by overwhelmed ICU services owing to exponential surges in the number of cases of COVID-19.

Japan experienced its first wave of patients with COVID-19 beginning in late March, which reached its peak in late April. However, the total number of patients at the peak in Japan was relatively small. On April 27, a maximum of 10,211 patients were treated in a single day nationwide, and a total of 407 patients (4%) nationwide were receiving or had received mechanical ventilation (captured using CRISIS [CRoss Icu Searchable Information System] by the national registry, Japan ECMOnet for COVID-19 [4, 5]) (Fig. 1). These totals accounted for about one-third of the critical care beds available specifically for COVID-19 care. Interestingly, 288 patients (60%) were weaned from the ventilator and survived, 79 died, and 114 were still receiving mechanical ventilation on May 16, 2020 (3 weeks after the peak, Fig. 1); the expected mortality rates were between 16 and 48%, calculated by assuming all patients remaining on a ventilator will survive or die. Moreover, veno-venous extracorporeal membrane oxygenation (ECMO) was performed in 159 patients up to May 16. Of these, 92 patients were weaned from ECMO, 33 died, and 34 continued to receive ECMO, with expected mortality between 20 and 42%.

**Fig. 1:**
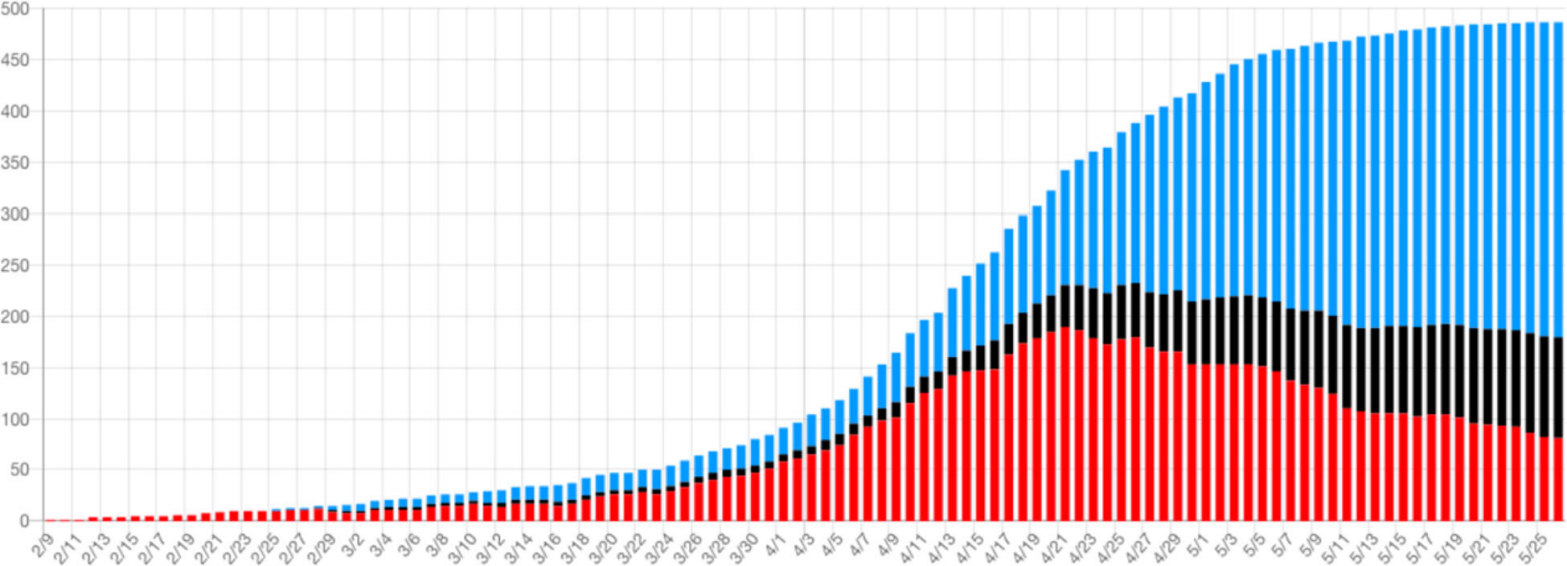
Cumulative cases daily captured using CRISIS [CRoss Icu Searchable Information System] by the national registry, Japan ECMOnet for COVID-19. Blue bars, survived and weaned off; black bars, died; red bars, remained on ventilator. *Y*-axis, number of cases; *X*-axis, dates in the year 2020

Although all the aforementioned studies are preliminary reports, that is, a considerable number of patients were still in the ICU on a ventilator, a relatively low mortality outcome is expected in the Japanese results. This suggests that reducing a “surge” in cases, which can overwhelm the capacity of the ICU, is critical for maintaining ordinary ICU functions, avoiding life-and-death triage decisions, and ultimately, saving the lives of patients in the face of this novel threat. More detailed scientific analysis and careful explanation of the data will be needed, considering the indications; patient background including age, comorbidities, or acute illness severity; and details of ventilator management, all factors that can considerably affect survival outcomes. In addition, analyses of the social factors contributing to flattening of the peak itself are urgently required as such findings could offer important insight into nationwide protective strategies against possible “next waves” of the COVID-19 pandemic in countries worldwide.

## Data Availability

The datasets used and analyzed during the current study are available from the corresponding author on reasonable request.

## Abbreviations

COVID-19: Coronavirus disease 2019
CRISIS: CRoss Icu Searchable Information System
ECMO: Extracorporeal membrane oxygenation
ICU: Intensive care unit
SARS-CoV-2: Severe acute respiratory syndrome coronavirus 2
